# Understanding non-recreational prescription medication-sharing behaviours: a systematic review

**DOI:** 10.3399/BJGP.2023.0189

**Published:** 2024-02-20

**Authors:** Shoba Dawson, Hans Johnson, Alyson L Huntley, Katrina M Turner, Deborah McCahon

**Affiliations:** Sheffield Centre for Health and Related Research, University of Sheffield, Sheffield.; Bristol Medical School (Population Health Sciences), University of Bristol, Bristol.; Centre for Academic Primary Care, Bristol Medical School (Population Health Sciences), University of Bristol, Bristol.; Centre for Academic Primary Care, Bristol Medical School (Population Health Sciences), University of Bristol, Bristol; National Institute for Health Research Applied Research Collaboration West (NIHR ARC West) at University Hospitals Bristol and Weston NHS Foundation Trust, Bristol and NIHR Bristol Biomedical Research Centre, University Hospitals Bristol NHS Foundation Trust and University of Bristol, Bristol.; Centre for Academic Primary Care, Bristol Medical School (Population Health Sciences), University of Bristol, Bristol.

**Keywords:** borrowing, general practice, loaning, prescription medicines, primary care, sharing, systematic review

## Abstract

**Background:**

Prescription medication sharing refers to the lending or borrowing of prescription medications where the recipient is someone other than the person for whom the prescription is intended. Sharing prescription medication can cause significant harm. Adverse consequences include an increased risk of side effects, delayed health seeking, and severity of disease. Prevalence estimates vary across different populations and people’s reasons for, and perceptions of risks from, sharing are poorly understood.

**Aim:**

To better understand prescription medication-sharing behaviours and practices – specifically, the prevalence, types of medications, reasons, perceived benefits and risks, and factors associated with medication sharing.

**Design and setting:**

This systematic review included primary studies in any setting, focusing on people who engage in medication sharing.

**Method:**

Electronic databases were searched from inception of databases to February 2023.

**Results:**

In total, 19 studies were included. Prevalence of lifetime sharing ranged from 13% to 78%. All 19 studies reported that analgesics were the most shared, followed by antibiotics (*n* = 12) and allergy medication (*n* = 9). Common reasons for sharing were running out of medication (*n* = 7), cost (*n* = 7), and emergency (*n* = 6). Perceived benefits included resolution of the problem and convenience. Perceived risks included adverse drug reactions and misdiagnosis. Characteristics associated with sharing included age, female sex, having asthma, and unused medicines stored at home.

**Conclusion:**

Findings suggest that medication-sharing behaviour is common and involves a range of medicines for a variety of reasons. Data on the prevalence and predictors of prescription medication sharing are inconsistent. A better understanding of non-modifiable and potentially modifiable behavioural factors that contribute to sharing is needed to support development of effective interventions aimed at mitigating unsafe sharing practices.

## Introduction

Prescribing of medicines is the major therapeutic intervention available to clinicians, with the majority occurring in primary care.^1^^,^^2^ When taken as prescribed, medicines have the potential to improve health and quality of life. However, if not taken correctly, medicines can be associated with harm. The World Health Organization estimates that half of all patients fail to take prescription medicines correctly.^3^ The inappropriate use of prescription medicines has significant resource implications for the health service and is associated with a range of adverse health consequences for patients.^4^

Sharing of prescription medicines is a form of inappropriate medication use. Medication sharing is defined as the lending or borrowing of prescription medicines where the recipient of those medicines is someone other than the person for whom the prescription is intended.^5^^,^^6^ Non-recreational sharing of prescription medication (for example, sharing for medical use) can cause significant harm to the individuals who engage in these practices. Loss of counselling and instruction from prescribers and dispensers in primary care (such as general practices and community pharmacies) when sharing prescription medicines can lead to an increased risk of side effects. For example, for females of reproductive age, the consequences of borrowing antibiotics may include decreased effectiveness of oral contraception and exposure to isotretinoin when borrowing treatment for severe acne can cause congenital malformation.^5^ Other potential adverse consequences include treatment failure, delayed health-seeking behaviour, masking of the symptoms and severity of disease, and antibiotic resistance.^7^^–^10

**Table 1:** How this fits in

Sharing of prescription medicines for non-recreational purposes is a form of inappropriate medication use and such practices can cause delays in seeking medical care, masking the symptoms and severity of disease, and could potentially result in the progression of the health condition. The reasons why people engage in medication sharing, how they assess the potential risks and benefits of these practices, and the factors that influence these behaviours are poorly understood. This systematic review shows that prescription medication sharing for non-recreational purposes is common, with analgesics and antibiotics being the most shared medications; however, data on the prevalence and predictors of these behaviours are limited. This review highlights that prescription medication sharing for non-recreational purposes is a potentially important medicines safety issue and significant public health concern that merits healthcare provider intervention, public awareness efforts, and further research.

Prescription medication-sharing behaviour is highly relevant to primary healthcare providers because it has an impact on patient safety and can disrupt medication adherence, continuity of care with respect to treatment planning and monitoring, and efforts to combat antimicrobial resistance. The first review to systematically summarise research on non-recreational medication sharing was published in 2014. The review reported prevalence rates for borrowing prescription medication ranging from 5% to 52% and for loaning from 6% to 23%.^7^ The authors concluded that there is insufficient data on reasons for medication sharing, how patients decide to engage in medication sharing, and whether they are aware of the potential risks.

Over the past few decades, several changes in drug classification from prescription-only to non-prescription medicine (for example, over the counter) have occurred and initiatives to improve self-care and access to non-prescription medicines have been heavily promoted within health policies nationally and internationally.^11^^,^^12^ As such, the types of medication shared, reasons for sharing, and perceptions of the potential harms associated with prescription medication have likely changed. For this reason, the aim of the current systematic review was to examine current evidence to:

- better understand the prevalence and types of prescription medications shared;
- the reasons why people share;
- who people share with;
- what the consequences of prescription medication sharing are; and
- the potential risk factors for sharing behaviour.

## Method

### Search strategy

The reporting of this review follows the PRISMA guidelines.^13^ The protocol was registered on PROSPERO (CRD42021252209). MEDLINE, Embase, CINAHL, Cochrane Library, and PsycINFO were searched from inception to February 2023. A search strategy was developed combining MeSH and free-text terms (Supplementary Box S1).^7^

### Inclusion and exclusion criteria

People in any setting and any age were included. Primary studies of any design examining prescription medication sharing (for example, lending and/or borrowing) for non-recreational purposes from the perspective of those who engage in the behaviour were included. Studies on sharing of prescription medication for recreational purposes, sharing of non-prescription medicines, or healthcare professionals’ perceptions of why people engage in these practices were excluded. Studies that did not primarily focus on medication-sharing behaviours and practices were also excluded. Editorials, policy briefings, books, book chapters, case reports, letters/opinion pieces, and systematic reviews were excluded. The reference lists of systematic reviews that met the criteria were searched to identify relevant primary studies. There were no language restrictions, provided an English-language abstract is available.

### Study selection

Rayyan^14^ was used to combine and screen the search results after deduplication. Two reviewers independently screened the titles/abstracts against the inclusion criteria. This was followed by full text. Any disagreements were resolved through including a third reviewer.

### Data extraction, quality appraisal, and synthesis

A data extraction proforma was tested on two randomly selected studies and refined. Data extracted were author, year, country, study design, population, prevalence of sharing, frequency, types of medicines, reasons for sharing, and consequences. Data were also extracted for PROGRESS-Plus dimensions:^15^ place of residence, race/ethnicity/culture/language, occupation, gender or sex, religion, education, socioeconomic status, and social capital. The Plus element includes factors that can affect health equity such as age, disability, and sexual orientation. As multiple designs were included, the Mixed Methods Appraisal Tool (MMAT)^16^ was used to critically appraise included studies. One author extracted data and completed the MMAT, and two authors checked and reconciled them. As a result of the heterogeneous characteristics of the included studies, a narrative approach was used to analyse and synthesise the findings using descriptive text and tables to summarise data. Within the tables, loaning and borrowing behaviours are presented separately where it was possible to distinguish which type of sharing was explored.

## Results

After deduplication 3503 titles/abstracts were screened. Fifty-three full texts were screened for eligibility and 19 studies reported in 23 publications were included (Supplementary Figure S1).^5^^,^^8^^,^^9^^,^^17^^–^36

### Characteristics of included studies

Seven studies were from the US (eight papers),^5^^,^^9^^,^^17^^–^21^,^^36^ three from New Zealand (four papers),^8^^,^^22^^–^24 one study each from Croatia (three papers),^25^^–^27 Saudi Arabia,^28^ Malaysia,^29^ Nigeria,^30^ Philippines,^31^ Australia,^32^ Ireland,^33^ South Korea,^34^ and Uganda.^35^ Seventeen studies employed quantitative methods,^5^^,^^9^^,^^17^^–^31^,^^33^^–^36 one was qualitative,^8^ and one mixed methods.^32^

Thirteen studies included only adults,^8^^,^^9^^,^^18^^,^^22^^,^^23^^,^^25^^–^29^,^^31^^–^36 two studies included only young people aged ≤18 years,^5^^,^^17^ and three included adults and young people.^20^^,^^21^^,^^24^^,^^30^ One study included caregivers with children <17 years.^19^ Fifteen studies had ≥50% females, with one study not reporting participants’ sex.^36^ Eleven studies^8^^,^^9^^,^^17^^–^24^,^^28^^,^^29^^,^^36^ reported participants’ ethnicity and/or race. Sample size ranged from 17^8^ to 26 289^36^ participants (Supplementary Table S1).

### PROGRESS-Plus

All 19 studies (100%) considered at least one element of PROGRESS-Plus when describing study participants (Supplementary Table S2). Of the 19 studies, 13 (68%) described at least one element within the research question. Age and place of residence were the most reported item (100%), followed by gender (95%, 18/19) and education (89%, 17/19). Other reported items included race/ethnicity/language/culture (58%, 11/19), occupation (53%, 10/19), and socioeconomic status comprising at least one of the following: household income, housing status, health literacy, insurance, (53%, 10/19), social capital, comprising marital status and/or household size (47%, 9/19), and religion (5%, 1/19).

Only 16 studies (84%), analysed outcomes by one element with most included being age and gender/sex (16/19, 84%), education (10/19, 53%), social capital (9/19, 47%), socioeconomic status (8/19, 42%), race/ethnicity/language spoken (6/19, 32%), and employment (5/19, 26%).

### Methodological quality

The qualitative and quantitative descriptive components were of high quality and the mixed-methods component was of moderate quality (Supplementary Table S3).

All studies had defined research questions addressed by the data collected. The qualitative study component showed coherence between data sources, collection, analysis, and interpretation.^8^ All quantitative studies used appropriate sampling with two studies having a sample representative of the target population;^5^^,^^36^ while remaining studies did not have a representative sample.^9^^,^^17^^–^31^,^^33^^–^35 All the studies used appropriate measurements to assess outcomes and analysis.^5^^,^^9^^,^^17^^–^31^,^^33^^–^36 The risk of non-response bias was high as this was not assessed within the included studies.^5^^,^^9^^,^^17^^–^31^,^^33^^–^36 The mixed-methods component showed adequate rationale for the chosen method, integration of mixed-methods components, and met the quality criteria.^32^ However, it was unclear if the integration of outputs was adequately interpreted, or if divergence between components was adequately addressed.^32^

### Types of medications shared

Two studies did not report the type of medication shared.^8^^,^^29^ Of the studies that provided details:

- fifteen studies reported that painkillers were commonly shared.^9^^,^^17^^–^28^,^^30^^,^^32^^,^^34^^–^36
- twelve antibiotics;^9^^,^^18^^–^24^,^^28^^,^^30^^,^^31^^,^^33^^–^35
- nine allergy medication;^18^^–^24^,^^28^^,^^34^^–^36
- five antidepressants/anxiety/mood medication;^9^^,^^18^^,^^20^^,^^21^^,^^30^^,^^32^
- three medications for skin problems and acne;^18^^,^^20^^,^^21^^,^^30^
- three contraceptives;^18^^,^^20^^,^^21^^,^^33^
- three sleeping medication/sedatives;^5^^,^^17^^,^^36^
- two asthma medications;^19^^,^^22^^,^^23^
- two blood pressure medication;^9^^,^^32^ and
- only one study reported the prevalence of sharing each of heart disease medication,^32^ diabetes medication,^32^ arthritis/joint inflammation medication,^32^ cold/flu medication,^30^ and stimulants.^17^

### Prevalence of loaning, borrowing, or sharing

Prevalence for borrowing or loaning ranged from 14% to 54% and lifetime sharing ranged from 13% to 78% (Supplementary Table S4). One study reported future sharing/loaning (69%) and borrowing (78%).^23^

People shared medication with family members (87%);^34^ friends only (26%);^24^ neighbours only (63%);^35^ friends and/or acquaintances (7%);^25^^–^27 and colleagues (8%).^25^^–^27 Also, 9% borrowed from family members^30^ and 75% from neighbours.^35^ Five studies did not report on the relationship between medication sharers.^8^^,^^18^^,^^23^^,^^28^^,^^32^

### Reasons for sharing

People shared medication for a variety of reasons (Supplementary Table S4). Many of the reasons were related to situations and circumstances in which sharing occurred or opportunities that led to sharing rather than the motivation for the behaviour itself (Supplementary Table S4). As a result of this in the current study reported reasons have been categorised as:

- motivation for sharing;
- situations/circumstances where sharing occurred; and
- opportunities for sharing.

The most common motivation for sharing was avoidance of the cost associated with visiting a medical professional and/or obtaining prescription medication (*n* = 7). 5^,^^9^^,^^19^^,^^20^^,^^32^^,^^33^^,^^36^ This was followed by not wanting to visit a medical professional (*n* = 6)^18^^,^^19^^,^^21^^,^^28^^,^^33^^,^^34^ and to access medicines/medical services when needed (*n* = 5).^5^^,^^8^^,^^21^^,^^25^^,^^27^^,^^34^

Running out of medication was the most commonly reported circumstance(*n* = 7),^5^^,^^19^^,^^20^^,^^24^^,^^28^^,^^30^^,^^36^ followed by requiring urgent/emergency treatment (*n* = 6).^5^^,^^19^^,^^20^^,^^24^^,^^30^^,^^34^

Several studies identified opportunities that enabled medication sharing. Commonly reported opportunities were having a similar problems/illness to another person who was in possession of a prescription medication to treat that condition (*n* = 5);^19^^,^^24^^,^^28^^,^^30^^,^^36^ followed by availability of medication because of another person having unused medication stored at home (*n* = 6),^5^^,^^8^^,^^19^^,^^20^^,^^30^^,^^36^ and convenience (*n* = 3).^9^^,^^25^^,^^27^^,^^33^

Four studies did not assess the reasons for sharing.^17^^,^^29^^,^^31^^,^^35^ From the current review of included qualitative studies, reasons included limited access to medical services when in need of medicines and/or distrust in healthcare professionals because of prior negative experiences. Availability of unused medicines or medicines prescribed to friends and close family were cited as common reasons for borrowing prescription medication.^8^^,^^25^ These reasons for borrowing are likely factors that influence delay in seeking medical help.

### Perceived benefits and risks of sharing

Seven studies reported on perceived risks and benefits of sharing.^8^^,^^18^^,^^21^^–^23^,^^25^^–^27^,^^30^^,^^34^ Only three of them described people’s perceptions of the benefits and risks of prescription medication sharing.^8^^,^^25^^,^^27^^,^^30^ Benefits included:

- improve health;^30^
- saving time and money (avoidance of a visit to doctor and associated costs, convenience);^8^^,^^25^^–^27 and
- maintaining good relationships with friends and co-workers.^25^^–^27

In the four studies where responders were asked if they experienced a side effect, between 2.3% and 37% reported experiencing side effects after borrowing prescription medication.^18^^,^^21^^–^23^,^^30^ Other risks included no problem resolution;^30^ risk of harm, an adverse drug reaction, and misdiagnosis;^8^^,^^25^^–^27 detrimental effects on interpersonal relationships;^25^^,^^27^ use of an incorrect dosage and loss of medication instructions^8^^,^^25^^–^27 (Supplementary Table S5).

Twelve studies did not assess the perceived benefits and risks of sharing medicines.^5^^,^^9^^,^^17^^,^^19^^,^^24^^,^^28^^,^^29^^,^^31^^–^33^,^^35^^,^^36^ Only one identified risks to public health and possible drug dependency as a consequence of sharing.^8^

### Predictors of prescription medication-sharing behaviour

Non-modifiable factors associated with increased likelihood of medication sharing included female sex,^35^ younger age,^24^^,^^35^^,^^36^ larger household size, and a prescription for asthma medication.^20^^,^^21^^,^^23^^–^24 Having unused or stored medication was also associated with an increased likelihood of sharing or loaning.^24^ One quantitative study indicated an association between older age and loaning behaviour.^23^

Predictors of borrowing behaviour were:

- younger age;^25^^,^^27^^,^^30^^,^^35^
- female sex;^35^
- a prescription for asthma medication;^23^ and
- storage of unused medicines.^24^^,^^28^

## Discussion

### Summary

Overall findings suggest that prescription medication sharing for non-recreational purposes is common and involves diverse classes of medication across different countries within which healthcare systems may be publicly or privately funded. Over 50% of the studies were from developed countries, highlighting a lack of research from developing countries where essential medicines may be less available, affordable, and/or accessible.^37^ The prevalence of loaning ranged from 6% to 67%, whereas borrowing ranged from 5% to 56%. Lifetime sharing ranged from 13% to 78%.

The current review also shows that painkillers and allergy medications are commonly shared classes of medication. This may be because people are more comfortable to report sharing of medicines that are not exclusively available on prescription (for example, can be purchased over the counter). Furthermore, people may perceive these types of medicines as low risk or safer to share than medicines that are only available via prescription. However, several studies reported a significant minority of responders had shared antibiotics and mood/antidepressants that are generally only prescription medicines. This suggests that some people were willing to disclose their sharing behaviours and their responses were not subject to social desirability bias.

Where studies assessed perceived risks/benefits of sharing, however, a range of risks and only a few benefits were identified. Benefits identified included improvement of the health problem, saving time and money, and maintaining good relationships. Identified risks included sharing practices being unsafe and borrowed medicines being considered ineffective, concerns about side effects and adverse drug reactions, potential for drug dependency, and risks to public health.

Reasons for sharing were multifactorial, encompassing motivations and opportunities for sharing alongside situations in which sharing often occurred. Motivations were predominantly related to problems accessing medicines and medical services when they were most needed. Similarly, medication sharing occurred in emergency situations or when people had run out of medicines. Storage of unused medication in the home provided people with an opportunity to share.

### Strengths and limitations

The main strength of this review is that it provides a comprehensive overview of the published literature on this topic area and highlights gaps in knowledge. Other strengths include registration of the protocol with PROSPERO, to promote transparency and replicability. Additionally, in accordance with calls for improving the assessment and reporting of health equity in research, the PROGRESS-Plus framework was used to provide an overview of health-equity-related information in this topic area.

There were, however, limitations. Differences in healthcare systems, definitions for, and measures of medication sharing across studies made drawing conclusions challenging. Loaning and borrowing are reported on separately throughout the current paper where possible and the term ‘sharing’ has been used where there is ambiguity with respect to the behaviour being assessed and reported within studies. Since data were inconsistent, it was not possible to reliably identify any non-modifiable risk factors (for example, age, sex, specific medical condition) or potentially modifiable predictive factors (for example, perceptions of harm and benefit, difficulty accessing medicines/services) associated with medication-sharing behaviour. Nevertheless, the current review provides helpful insights into the prevalence of, and reasons for, prescription medication sharing across different settings and suggests that prescription medication sharing is a common and potentially important medicines safety issue.

### Comparison with existing literature

Like the previous review,^7^ in the current study it was noted there were limited data for medication sharing and ethnicity and race because of poor reporting of participant characteristics. Although this information was collected as part of demographics, it was not used in analysis, so the influence of ethnicity and race has not been studied thoroughly. There is evidence from other studies that prescription drug use varies by race and country of birth, so it is likely that prescription medication behaviours and practices also vary in this respect.^38^

The prevalence of loaning and lifetime sharing was, however, much higher in the current review than the previous review.^7^ This is perhaps unsurprising given that over the past decade several changes in drug classification have occurred and initiatives for self-medication have been heavily promoted.^39^ These policy changes are likely to have influenced people’s safety perceptions for both prescription-only and pharmacy medicines, and potentially encouraged prescription medication-sharing practices.

There is evidence from a recent scoping review that during public health emergencies, such as pandemics or natural disasters, individuals without medical training self-alter their prescribed medication regimen as a means of coping with challenges related to the availability and accessibility of healthcare services.^40^ Two studies included within the scoping review reported adaptive medication practices that included sharing of prescription medications such as insulin and buprenorphine for non-recreational purposes following Hurricanes Sandy and Katrina.^41^^,^^42^ Furthermore, a recent UK-based qualitative study indicated that rapid digitalisation of primary care in response to the pandemic has exacerbated existing inequalities regarding access to care for some groups of the population through a lack of availability or knowledge of technology.^43^ As such, it is possible that reduced access to healthcare services during the COVID-19 pandemic may have increased the prevalence of prescription medication sharing in more recent years. Initiatives to improving access to prescription medication and return of unused medicines may help reduce the likelihood of sharing.

### Implications for research and practice

Complete elimination of prescription medicine sharing is likely to be unachievable and may be undesirable, given that, in some situations, sharing can improve health outcomes.^44^^,^^45^ For example, having immediate access to appropriate medication during anaphylaxis, an acute asthma exacerbation, or an opioid overdose can be lifesaving. As such, a harm mitigation approach to addressing unsafe medication-sharing practices is warranted.

Globally, sharing of analgesics and antibiotics appears to be particularly common and therefore is a public health concern that merits healthcare provider attention alongside public awareness efforts. Healthcare providers can help to reduce the perceived need and opportunity for sharing of such medicines through education about appropriate medication use, disposal, and the availability of alternative options for obtaining timely access to medicines.

Future research should focus on the identification of those most likely to engage in unsafe medication-sharing practices. This is important to help healthcare providers deliver targeted interventions aimed at reducing potential harms. A better understanding of the potentially modifiable behavioural, social, and psychological factors that contribute to unsafe prescription medication-sharing practices is, however, also required to inform the development of engaging, persuasive, and effective interventions. To help determine the actions required to mitigate unsafe medication-sharing practices and associated harms, researchers should consider using qualitative research methods to explore both patient and healthcare provider experiences and perspectives of medication-sharing behaviour.

Sharing behaviours within racial and ethnic minority groups are not well characterised within the literature. It is widely known, however, that these groups experience poorer access to health care and health outcomes, including higher rates of hospital-acquired infections, complications, adverse drug events, and dosing errors when compared with the wider population.^46^ There is also mistrust in healthcare professionals. All these factors coupled with intersectional issues, such as socioeconomic status, education, and religion, may contribute towards increased and potentially unsafe lending/borrowing behaviour. Research is required to understand the unique medication-related challenges faced by these groups and promote equitable and safe medicines use practices.

In conclusion, this review has highlighted that prescription medication-sharing behaviour is common with a range of medications being shared for a variety of reasons. Evidence to support identification of those most likely to engage in prescription medication-sharing practices is inconsistent and insufficient. Further research is also needed to better understand the potentially modifiable behavioural, social, and psychological factors that contribute to sharing and support the development of effective interventions aimed at mitigating unsafe sharing practices and associated harms.

## Data

These are publicly available papers as they are all published. Extracted data are available as tables in the supplementary information for this article.
